# A Novel Rabbit Immunospot Array Assay on a Chip Allows for the Rapid Generation of Rabbit Monoclonal Antibodies with High Affinity

**DOI:** 10.1371/journal.pone.0052383

**Published:** 2012-12-26

**Authors:** Tatsuhiko Ozawa, Xiuhong Piao, Eiji Kobayashi, Yue Zhou, Hiroaki Sakurai, Tsugunobu Andoh, Aishun Jin, Hiroyuki Kishi, Atsushi Muraguchi

**Affiliations:** 1 Department of Immunology, Graduate School of Medicine and Pharmaceutical Sciences, University of Toyama, Toyama, Japan; 2 Department of Cancer Cell Biology, Graduate School of Medicine and Pharmaceutical Sciences, University of Toyama, Toyama, Japan; 3 Department of Applied Pharmacology, Graduate School of Medicine and Pharmaceutical Sciences, University of Toyama, Toyama, Japan; 4 Department of Immunology, College of Basic Medical Sciences, Harbin Medical University, Nangang District, Harbin, China; Cordelier Research Center, INSERMU872-Team16, France

## Abstract

Antigen-specific rabbit monoclonal antibodies (RaMoAbs) are useful due to their high specificity and high affinity, and the establishment of a comprehensive and rapid RaMoAb generation system has been highly anticipated. Here, we present a novel system using immunospot array assay on a chip (ISAAC) technology in which we detect and retrieve antigen-specific antibody-secreting cells from the peripheral blood lymphocytes of antigen-immunized rabbits and produce antigen-specific RaMoAbs with 10^–12^ M affinity within a time period of only 7 days. We have used this system to efficiently generate RaMoAbs that are specific to a phosphorylated signal-transducing molecule. Our system provides a new method for the comprehensive and rapid production of RaMoAbs, which may contribute to laboratory research and clinical applications.

## Introduction

Monoclonal antibodies are widely used in laboratory research as well as clinical applications due to their high specificity and high affinity. Notably, compared to conventional rodent antibodies, rabbit monoclonal antibodies (RaMoAbs) are ideal for investigation and diagnosis for two reasons. First, rabbit antibodies generally exhibit a high affinity and high specificity [1]–[6]. Second, rabbits are known to produce antibodies to many antigens that are not immunogenic in mice or other animals [1], [4], [7]–[11].

Mouse-rabbit hetero-hybridomas were initially used to produce RaMoAbs [10], [12]–[14]. However, these hetero-hybridomas were highly unstable, difficult to clone, and unable to secrete antibodies for prolonged periods [2]. In 1995, Knight and colleagues established a plasmacytoma cell line over-expressing v-*abl* and c-*myc*, which they used as a hybridoma partner cell line [6], [15]. Nevertheless, the hybridoma method is not widely used at the laboratory level.

We have established a rapid, efficient, and high-throughput system for identifying and recovering objective antibody-secreting cells (ASCs) using microwell array chips and immunospot array assay on a chip (ISAAC) technology [16], [17]. Microwell array chip has an array of up to 234,000 wells, and each well has a size and shape that are optimized for the accommodation of a single lymphocyte; this feature has enabled us to analyze live cells on a single-cell basis. The ISAAC system can detect antigen-specific ASCs in human peripheral blood lymphocytes (PBLs) and can produce antigen-specific human monoclonal antibodies within 7 days.

In this study, we used the ISAAC system to detect antigen-specific antibody-secreting single primary B-cells from rabbits. We demonstrated that the rabbit-ISAAC system allows for the comprehensive and rapid production of RaMoAbs with high affinity. Moreover, the system can produce RaMoAbs that are specific to a phosphorylated signal-transducing molecule. This innovative technology may contribute to the high-throughput production of RaMoAbs for laboratory research and clinical applications.

## Results

### The Rabbit-ISAAC System

**Figure 1:**
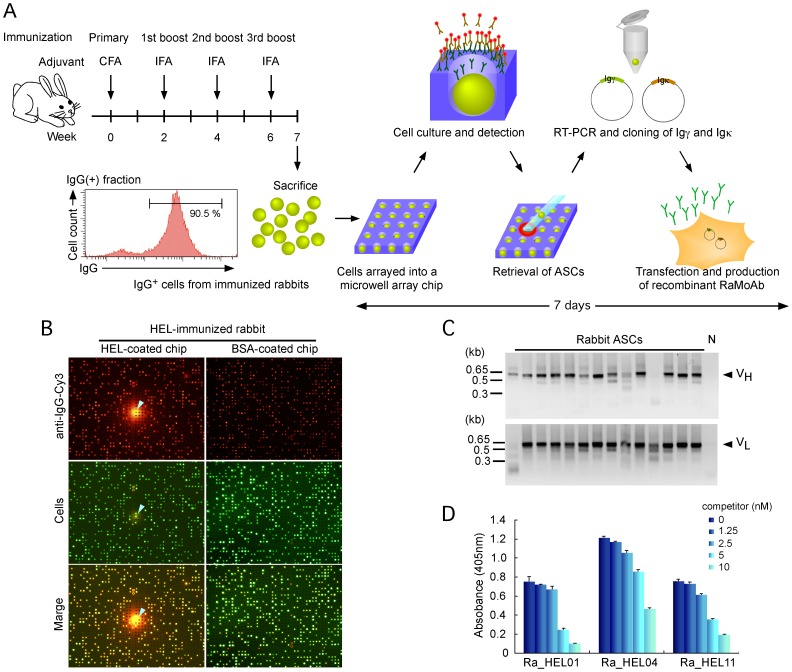
The rabbit-ISAAC system. ( A) A scheme depicting the rabbit-ISAAC procedure. Briefly, rabbits were immunized and later sacrificed 1 week after the final boost. Rabbit IgG^+^ cells were sorted, and single antigen-specific ASCs were detected using a microwell array chip. Next, rabbit immunoglobulin cDNAs were amplified from single cells, cloned into expression vectors and co-transduced into CHO-S cells. The antigen specificity of the produced antibodies can be determined 7 days after the detection of antigen-specific ASCs on the microwell array chip. (B) Detection of single HEL-specific ASCs in PBLs from HEL-immunized rabbits by the rabbit-ISAAC technique. Antibody secretion was detected with HEL (top left) or BSA (negative control, top right). The cells were then stained with Oregon Green (middle), and the antibody immunospots and cell signals were merged (bottom). Arrowheads indicate HEL-specific ASCs. (C) Amplification of rabbit V_H_ (top) and V_L_ (bottom) with a single-cell 5′-RACE procedure. The PCR products were analyzed by agarose gel electrophoresis. Sizes are shown at the left. Each lane shows the 5′-RACE products of individual cells. “N” indicates a cell-free RT-PCR reaction. (D) Competitive ELISA of HEL-specific RaMoAbs. The antigen specificity of antibodies derived from single ASCs using ISAAC was examined by competitive ELISA using varying amounts of HEL. The data represent means ± s.d. of 3 independent experiments.

To establish the rabbit-ISAAC system, we first tested the feasibility of the ISAAC system for detecting rabbit ASCs on a single-cell basis. We immunized a rabbit with hen egg lysozyme (HEL) and prepared IgG^+^ lymphocytes from PBLs (Figure 1A). We then arrayed IgG^+^ cells on a HEL-coated chip and detected HEL-specific ASCs using a Cy3-conjugated antibody that was specific to rabbit IgG. The secreted antibodies produced very strong immunospots on the HEL-coated chip that were not observed on the BSA-coated chip (Figure 1B).

We then retrieved 189 single cells that produced HEL-specific IgG immunospots and transferred the individual cells into separate micro-tubes for the amplification of cDNAs encoding the immunoglobulin heavy (H) and light (L) chain variable regions (V_H_ and V_L_). We amplified V_H_ and V_L_ cDNAs using a single-cell 5′-RACE method [17]–[19] with primers for the γ chain and the κ chain (Figure 1C). We amplified 56 pairs of V_H_ and V_L_ cDNAs and inserted these into expression vectors that contained the cDNA of the rabbit immunoglobulin constant region (γ or κ chain). Thereafter, we co-transfected both the γ and κ chain expression vectors together in CHO-S cells, which then produced 55 RaMoAbs. An ELISA showed that 24 of 55 antibodies were specific to HEL (Figure 1D, Table 1, and Table S1).

**Table 1:** Characterization of HEL-specific RaMoAbs.

ID	IGHV	IGHD	IGHJ	IGKV	IGKJ	Frequency^a^	*K*D (M)^b^
Ra_HEL01	1S44	2	4	1S47	1–2	3	2.63×10^–12^
Ra_HEL04	1S44	6	4	1S37	1–2	2	7.00×10^–12^
Ra_HEL06	1S44	6	4	1S36	1–2	1	1.45×10^–12^
Ra_HEL07	1S44	6	4	1S36	1–2	1	3.14×10^–9^
Ra_HEL08	1S44	2	4	1S36	1–2	1	6.09×10^–10^
Ra_HEL09	1S21	1	4	1S36	1–2	1	4.15×10^–11^
Ra_HEL10	1S44	2	4	1S36	1–2	1	3.77×10^–12^
Ra_HEL11	1S44	2	4	1S36	1–2	1	3.93×10^–12^
Ra_HEL12	1S44	2	2	1S37	1–2	1	2.09×10^–7^
Ra_HEL13	1S44	2	4	1S64	1–2	1	5.77×10^–8^
Ra_HEL14	1S43	8	4	1S44	1–2	1	5.06×10^–11^
Ra_HEL15	1S44	1	4	1S12	1–2	1	8.07×10^–10^
Ra_HEL16	1S44	6	2	1S36	1–2	1	3.25×10^–10^
Ra_HEL17	1S44	1	2	1S4	1–2	1	7.12×10^–7^
Ra_HEL18	1S44	6	4	1S47	1–2	1	1.47×10^–12^
Ra_HEL19	1S44	2	4	1S42	1–2	1	1.69×10^–9^
Ra_HEL20	1S44	6	4	1S36	1–2	1	9.60×10^–11^
Ra_HEL21	1S44	2	4	1S36	1–2	1	1.35×10^–12^
Ra_HEL22	1S44	6	4	1S10	1–2	1	1.43×10^–11^
Ra_HEL23	1S44	4	4	1S4	1–2	1	1.72×10^–9^
Ra_HEL24	1S44	4	6	1S10	1–2	1	2.11×10^–9^

a Frequency indicates numbers of clones showing identical amino acid sequences.

b The data are presented as average of at least two experiments.

We then analyzed the cDNA sequences of 24 HEL-specific monoclonal antibodies and obtained 21 distinct sequences (Table 1). In agreement with the previous study [4], [20]–[22], all of these sequences contained a single IGVH1 gene, and a majority of the sequences contained J_H_4. Our results indicate that the antibody repertoire obtained from the analysis of primary rabbit ASCs using the rabbit-ISAAC system is similar to that obtained with the conventional hybridoma method.

We next measured the affinity of these antibodies by ELISA (Figure S1). The affinity (*K*D) for HEL ranged from 1×10^–7^ to 1×10^–12^ M (Table 1). Interestingly, the *K*D of 14 out of 24 (56%) antibodies for HEL was estimated to be 10^–10^ to 10^–12^ M. The RaMoAbs with extremely high affinity may detect very small amount of antigen. To examine this possibility, we determined the limit of detection (LOD) in ELISA for a RaMoAb with 10^–12^ M affinity (Ra_HEL21) and a mouse HEL-specific antibody (Mo_HEL10) that showed the highest affinity (3.71×10^–10^ M) among mouse HEL-specific antibodies obtained with ISAAC [17]. The calculated LOD of the RaMoAb was approximately 0.4 pM, and that of the mouse antibody was approximately 10 pM (Figure S2). The results demonstrated that the LOD of the RaMoAb of 10^–12^ M affinity was 25-fold lower than that of the mouse antibody.

### Relationship between Rabbit-ISAAC Immunospots and Affinities

**Figure 2:**
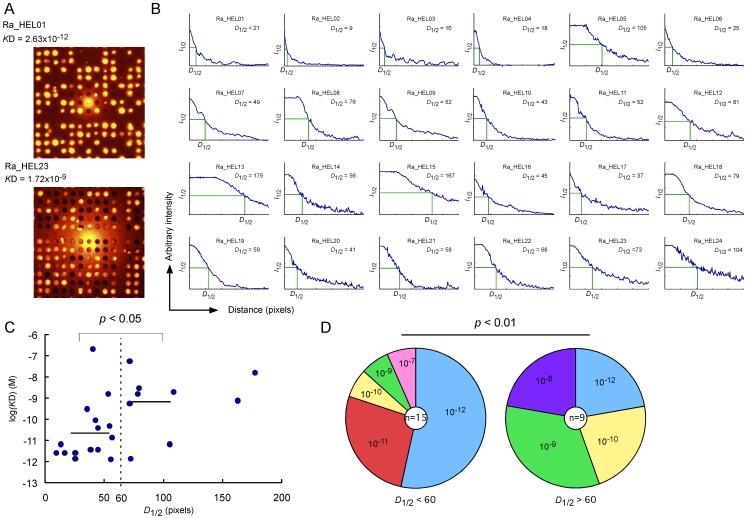
Relationship between rabbit-ISAAC immunospots and affinities. (A) Representative ISAAC immunospots of secreted HEL-binding RaMoAbs with high (*K*D: 10^−12^ M) and low (*K*D: 10^−9^ M) affinity. (B) A decay curve of fluorescence intensities in individual ISAAC immunospots. The fluorescence intensity of individual ISAAC immunospots (*y*-axis) is plotted against the distance (*x*-axis). *I*_1/2_ is the value at which the immunospot fluorescence intensity reaches 50%, and *D*_1/2_ indicates the distance at which *I*_1/2_ is achieved. The *D*_1/2_ value is shown in each individual plot. (C) Relationship between the *D*_1/2_ of individual immunospots (*x*-axis) and *K*D (*y*-axis). The dotted line indicates *D*_1/2_ = 60. The bar indicates the average *K*D for antibodies with *D*_1/2_<60 and those with *D*_1/2_>60. The *p*-value was determined using Student’s t-test. (D) Frequency of antibodies with the indicated order of *K*D values for antibodies with *D*_1/2_<60 and *D*_1/2_>60. The colored pie segment indicates the frequency of antibodies with the indicated order of *K*D. The number in the center of the pie chart denotes the number of antibodies analyzed. The *p*-value was determined using Fisher’s test.

The selection of antigen-specific antibodies with high affinity is one of the goals for the screening monoclonal antibodies. Adams *et al*. reported that antibodies with high affinity do not diffuse distantly in tumors [23]. Taking this into account, we predicted that rabbit-ISAAC immunospots of antibodies with high affinity might be smaller in diameter compared to immunospots of antibodies with low affinity. Thus, we measured the distance at which the fluorescence intensities of rabbit-ISAAC immunospots were 50% (*D*_1/2_) (Figure 2A and 2B). Next, we plotted the *D*_1/2_ and affinities of individual immunospots (Figure 2C). As expected, antibodies with higher affinities had smaller *D*_1/2_ values; the average *K*D of antibodies with immunospots of *D*_1/2_<60 was 2.56×10^–11^ M, while the *K*D of antibodies with *D*_1/2_>60 was 7.34×10^–10^ M (Student’s t-test, *p*<0.05) (Figure 2C). The *K*D of 12 out of 15 (80%) antibodies with *D*_1/2_<60 was between 10^–10^ and 10^–12^ M, while 2 out of 9 (22%) antibodies with *D*_1/2_>60 had *K*Ds of less than 10^–10^ M (Fisher’s test, *p*<0.01) (Figure 2D). These results indicate that antibodies with high affinities do not diffuse far, which is in agreement with Adams *et al*. [23]. Taken together, these findings demonstrate that we can obtain HEL-specific ASCs with high affinities by retrieving cells with *D*_1/2_<60; however, the *D*_1/2_ value may differ depending on the antigen as well as the antibody.

### An Efficient Screening System for Phosphorylated Peptide-specific RaMoAbs Using Rabbit-ISAAC

**Figure 3:**
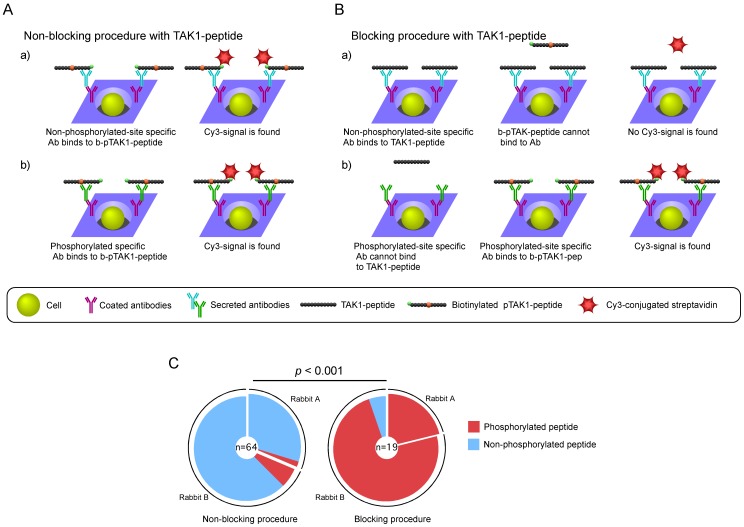
Efficient screening for phosphorylated peptide-specific RaMoAbs with rabbit-ISAAC. (A) Non-blocking procedure using the TAK1-peptide. Antibodies recognizing the non-phosphorylated peptide (a) and the phosphorylated peptide (b) can bind to biotinylated pTAK1-peptide, and a signal can be detected. (B) Blocking procedure using the TAK1-peptide. Microwell array chips were pre-treated with TAK1-peptide before the addition of biotinylated pTAK1-peptide. a) The TAK1-peptide binds to antibodies that recognize the non-phosphorylated site of the pTAK1-peptide. As a result, the biotinylated pTAK1-peptide cannot bind to the antibodies, and no signal is detected. b) The TAK1-peptide does not bind to antibodies that recognize the phosphorylated site of the pTAK1-peptide. As a result, the biotinylated pTAK1-peptide binds to antibodies, and a signal can be detected. (C) Acquisition efficiency of phosphorylated peptide-specific antibodies in the non-blocking and blocking procedures. The colored pie segment indicates the frequency of RaMoAbs that are specific to a phosphorylated peptide (red) and non-phosphorylated peptide (blue) in the non-blocking procedure (left) and the blocking procedure (right). The number in the center of the pie chart denotes the number of antibodies analyzed. The *p*-value was determined using Fisher’s test.

We next utilized the rabbit-ISAAC system to screen for ASCs producing antibodies that specifically recognize a phosphorylated peptide. We immunized rabbits with a phosphorylated peptide of human transforming growth factor-β-activated kinase 1 (TAK1), a key kinase regulating pro-inflammatory and innate/acquired immune signaling pathways [24]. Next, we prepared IgG^+^ cells from the PBLs of immunized rabbits and arrayed IgG^+^ cells on chips that were coated with rabbit IgG-specific antibodies. We attempted to detect Thr-187-phosphorylated TAK1, a critical site for TAK1 activation, (pTAK1)-peptide-specific ASCs using biotinylated pTAK1-peptide and Cy3-conjugated streptavidin (Figure 3). We obtained 64 monoclonal antibodies that bound to pTAK1-peptide. However, 59 of 64 monoclonal antibodies specifically bound not only to pTAK1-peptide but also to TAK1-peptide (Figure 3 and Table S1).

To efficiently screen for ASCs producing antibodies that specifically bind to pTAK1-peptide but not to TAK1-peptide, we first blocked the rabbit-ISAAC immunospots with TAK1-peptide. We then applied biotinylated pTAK1-peptide and Cy3-conjugated streptavidin to detect ASCs that were specific to pTAK1-peptide (Figure 3B). We obtained 19 monoclonal antibodies that bound to pTAK1-peptide. More than 90% (18 of 19) of the monoclonal antibodies specifically bound to pTAK1-peptide and not to TAK1-peptide (Figure 3C and Table S1). The acquisition efficiency of phosphorylated peptide-specific RaMoAbs was significantly improved compared to the non-blocking procedure (Fisher’s test, *p*<0.001) (Figure 3C). These results show that the use of the blocking procedure in the rabbit-ISAAC system is very useful for the isolation of monoclonal antibodies that specifically bind to pTAK1-peptide but not to TAK1-peptide.

We determined the affinity of these antibodies by ELISA (Figure S3). The affinity (*K*D) for pTAK1-peptide ranged from 1×10^–6^ to 1×10^–9^ M (Table 2). Sequencing analysis of the cDNA of 23 pTAK1-peptide-specific antibodies revealed eight distinct sequences (Table 2). All of these sequences contained a single IGVH1 gene, as previously reported [20]–[22]. Regarding J_H_, the frequency of J_H_4 usage was not so high as that of HEL antibodies.

**Table 2:** Characterization of pTAK1-peptide-specific RaMoAbs.

Rabbit	ID	IGHV	IGHD	IGHJ	IGKV	IGKJ	Frequency^a^	*K*D (M)^b^
A	Ra_pTAK01	1S44	1	3	1S19	1–2	3	6.13×10^–6^
A	Ra_pTAK04	1S44	7	2	1S56	1–2	1	2.76×10^–9^
A	Ra_pTAK05	1S44	4	3	1S2	1–2	1	3.26×10^–9^
B	Ra_pTAK06	1S44	7	4	1S2	1–2	8	1.15×10^–9^
B	Ra_pTAK14	1S44	8	4	1S46 or 60	1–2	5	2.30×10^–6^
B	Ra_pTAK19	1S44	1	2	1S12	1–2	2	2.06×10^–9^
B	Ra_pTAK21	1S44	1	2	1S52	1–2	2	5.64×10^–9^
B	Ra_pTAK23	1S44	7	4	1S2	1–2	1	1.23×10^–9^

a Frequency indicates numbers of clones showing the identical amino acid sequences.

b The data are presented as average of at least two experiments.

### Functional Characterization of pTAK1-peptide-specific RaMoAbs

**Figure 4:**
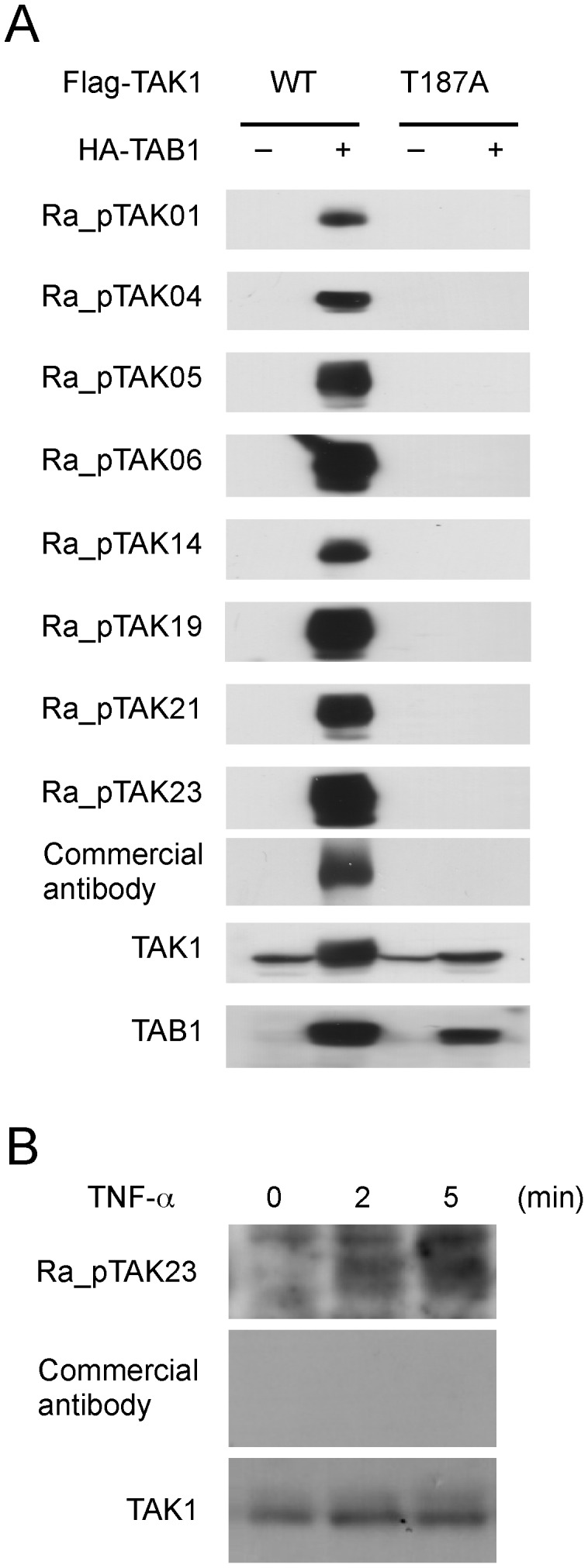
Western blot analysis of pTAK1-peptide-specific RaMoAbs. (A) Examination of the specificity of pTAK1-peptide-specific RaMoAbs. Whole-cell lysates obtained from HeLa cells that were transfected with plasmids containing FLAG-tagged wild type (WT) TAK1 or a phosphorylation site-substituted mutant (T187A) together with HA-tagged TAB1 were separated by SDS-PAGE and immunoblotted with Ra_pTAK01, 04, 05, 06, 14, 19, 21, and 23 antibodies, a commercial antibody specific to phosphorylated TAK1, a TAK1-specific antibody or a TAB1-specific antibody. (B) TNF-α-induced phosphorylation of endogenous TAK1 at Thr187. Whole cell lysates from HeLa cells that had been stimulated with 20 ng ml^−1^ TNF-α for the indicated time periods were separated by SDS-PAGE and immunoblotted with the Ra_pTAK23 antibody, a commercial pTAK1-pep-specific antibody, or a TAK1-specific antibody.

Finally, we characterized pTAK1-peptide-specific RaMoAbs using western blot analysis. It is known that over-expression of TAK1 with TAK1 binding protein 1 (TAB1) results in the phosphorylation of TAK1 at Thr-187 [25]–[27]. To examine whether pTAK1-peptide-specific RaMoAbs specifically detect Thr-187-phosphorylated TAK1, FLAG-tagged TAK1 and HA-tagged TAB1 were co-expressed in HeLa cells, and the cell lysates were immunoblotted with pTAK1-peptide-specific antibodies. The phosphorylation-specific band of TAK1 was clearly detected by co-expression with TAB1 (Figure 4A). In contrast, no phosphorylation was detected in the FLAG-tagged alanine-substituted mutant TAK1 (T187A) upon co-expression with TAB1 (Figure 4A).

We next examined whether our pTAK1-peptide-specific RaMoAb detects TNF-α-induced phosphorylation of endogenous TAK1. To this end, HeLa cells were stimulated with TNF-α; subsequently, the cell lysates were immunoblotted with pTAK1-peptide-specific antibodies. Immunoblotting with our Ra_pTAK23 antibody demonstrated that the bands could be detected in whole cell lysates from TNF-α-treated HeLa cells. Consistent with a previous study [25], the faster migrating bands corresponding to phosphorylated TAK1 were detected 2–5 min after the stimulation (Figure 4B). In contrast, a commercially available antibody could not detect the phosphorylation of endogenous TAK1 (Figure 4B). These results demonstrate that our RaMoAb recognizes Thr-187 phosphorylation of whole TAK1 molecules.

## Discussion

In this study, we have demonstrated a novel screening procedure for the production of RaMoAbs using the rabbit-ISAAC system (Figure 1). We demonstrated that the system efficiently produced RaMoAbs with high affinity (10^–10^ to 10^–12^ M) (Figure 2 and Table 1). We also demonstrated that the system could efficiently produce RaMoAbs that specifically recognize a phosphorylation-site-specific epitope (Figure 3 and Table 2).

In Table S1, we summarized the efficiency in each experimental step of rabbit ISAAC system. In the absence of blocking, from approximately 20 ml of blood, we obtained approximately 4×10^7^ PBL, and obtained 2×10^6^ IgG^+^ B cells. In the case of HEL, we analyzed a total of approximately 5.5×10^5^ IgG^+^ cells using 14 microwell array chips (62,500 wells, 15 µm in diameter). As a result, approximately 340 HEL-specific immunospots were detected, and 189 single cells were retrieved. From them, 56 pairs of V_H_ and V_L_ cDNAs were amplified and 55 RaMoAbs were produced. Finally, 24 of them specifically bound to HEL. In the case of pTAK1-peptide, a total of approximately 7.8×10^5^ IgG^+^ cells were analyzed using 24 microwell array chips. In all, approximately 590 TAK1/pTAK1-specific immunospots were detected, and 272 single cells were retrieved. From them, 128 pairs of V_H_ and V_L_ cDNAs were amplified and 118 RaMoAbs were produced. Finally, 64 of them specifically bound to TAK1/pTAK1 (Table S1). These results demonstrated the feasibility of ISAAC system for the analysis of antigen-specific ASC in rabbits and the comprehensive production of antigen-specific RaMoAbs.

A high affinity is one of the critical parameters for the selection of potent and efficacious therapeutic antibodies [28]–[31]. In this study, we analyzed the relationship between the sizes of the immunospots elicited by RaMoAbs produced by ASCs and their affinities. We found that the immunospots of antibodies with high affinity had smaller *D*_1/2_ values compared to immunospots of antibodies with low affinity (Figure 2C and 2D); this is likely because high affinity antibodies quickly bind to antigen near the well and do not diffuse distantly. This result is accordance with the study of Adams *et al.* [23], which showed that antibodies with high affinity do not diffuse distantly in tumors. These results illustrate that we can efficiently screen for ASCs that produce high affinity RaMoAbs by selecting ASCs that produce immunospots with smaller *D*_1/2_values. This screening procedure allows us to efficiently obtain antibodies with high affinities.

RaMoAbs with high affinities are useful for laboratory investigations and have many attractive features, including the ability to directly use reagents developed for western blot analysis that are substantially more sensitive. In this study, we showed that the RaMoAbs against HEL with 10^–12^ M affinity could detect 25-fold lower amount of the antigen compared to the mouse MoAbs that were obtained by mouse ISSAC (Figure S2). We also demonstrated that RaMoAb to pTAK1-peptide obtained by rabbit ISSAC detected endogenous phosphorylated TAK1 in TNFα-stimulated cells (Figure 4B). These data indicate that use of rabbit ISSAC system may contribute to the development of antibodies for not only laboratory use for clinical diagnosis but also researches in the field of signal transduction in cancer and inflammatory diseases, as well as Toll-like receptor signaling pathway.

We have demonstrated an efficient screening system for the procurement of phosphorylated peptide-specific ASCs using a blocking procedure (Figure 3). The acquisition efficiency was less than 10% using a non-blocking procedure; however, using a blocking procedure, the acquisition efficiency was extremely high (greater than 90%) (Figure 3). Thus, the rabbit-ISAAC system can be used to efficiently obtain a large panel of phosphorylation site-specific RaMoAbs, which may contribute to phosphoproteomic analyses in cell physiology [32], [33] and in tumorigenesis [34]. Furthermore, the blocking procedure used in the rabbit-ISAAC system may assist in obtaining antibodies that recognize subtle changes in epitopes or modified epitopes such as amino acid substitution, acetylation, or glycosylation.

In conclusion, the rabbit-ISAAC system is a groundbreaking technological advancement that produces RaMoAbs with high affinity and specificity within a time period of only 7 days. This system may greatly promote the high-throughput production of RaMoAbs for laboratory research and clinical applications.

## Materials and Methods

### Antigens and Peptides

We used hen egg lysozyme (HEL; Sigma), bovine serum albumin (BSA; Wako), human transforming growth factor-β-activated kinase 1 (TAK1)-peptide (TAK1-peptide, DIQTHMNNKGSAA; Operon Biotechnologies), TAK1-peptide phosphorylated at Thr-187 (pTAK1-peptide, DIQTHM(pT)NNKGSAA; Operon Biotechnologies), biotinylated pTAK1-peptide (DIQTHM(pT)NNKGSAAK-biotin; Operon Biotechnologies), and KLH conjugates of pTAK1-peptide (Operon Biotechnologies).

### Immunization of Rabbits and Cell Preparation

Experiments using rabbits were approved by the Committee on Animal Experiments at the University of Toyama. We immunized 12- to 13-week-old New Zealand White rabbits (Sankyo Lab) subcutaneously with 500 µg of HEL or KLH conjugates of pTAK1-peptide in complete Freund’s adjuvant (Millipore). Two, four, and six weeks after primary immunization, we boosted the rabbits subcutaneously with 500 µg of the same material used in the primary immunization in incomplete Freund’s adjuvant (Millipore). One week after the final boost, we isolated PBLs by centrifugation on a Ficoll–Hypaque gradient and isolated rabbit IgG^+^ cells with rabbit IgG-specific antibody-conjugated microbeads (Miltenyi Biotec) using an autoMACS Pro separator (Miltenyi Biotec) according to the manufacturer’s instructions.

### Detection of Rabbit ASCs with a Microwell Array Chip (ISAAC)

The ISAAC method is covered by patents that have been exclusively licensed to Vivalis (Nantes, France). Details and instructions regarding the microwell array chip and the ISAAC method have been previously described [16], [17], [35]–[38]. Briefly, to detect HEL-specific IgG secretion, we coated the surface of the chip with 10 µg ml^–1^ HEL in phosphate-buffered saline (PBS) and incubated it overnight at 4°C. After removing the antigen solution, we blocked the chip with 0.01% Biolipidure (NOF Corporation, Japan) for 15 min at room temperature and subsequently washed it with the culture medium. We then arrayed cells in culture medium to the chip and removed residual cells outside the wells with gentle washing. We cultured the cells on the chip for 3 h at 37°C. After gentle washing, we applied 1.5 µg ml^–1^ of Cy3-conjugated rabbit IgG-specific goat polyclonal antibody (Millipore) to the chip and incubated for 30 min at room temperature to detect antigen-specific IgG secretion. To detect pTAK1-peptide-specific IgG secretion, we coated the surface of the chip with 1 µg ml^–1^ of rabbit IgG-specific antibody (MP Biomedicals) to trap secreted IgG. After the cells were cultured on the chip for 3 h, we added 10 µg ml^–1^ biotinylated pTAK1-peptide and incubated for 30 min; this was followed by the addition of Cy3-conjugated streptavidin (Sigma) for 30 min. Where indicated, we added 10 µg ml^–1^ TAK1-peptide to the chip and incubated for 30 min before adding biotinylated pTAK1-peptide. Finally, we stained the cells with 1 µM Oregon Green (Molecular Probes) for 5 min at room temperature. Antigen-specific antibodies that were released from single cells were observed under a fluorescence microscope (BX51WI, Olympus).

### Production of RaMoAbs

We retrieved single antigen-specific ASCs from individual wells using a micromanipulator (TransferMan NK2, Eppendorf) fitted with capillaries (Primetech, Japan) under a fluorescence microscope and expelled them into micro-tubes containing a cell lysis solution composed of 30 µg of Dynabead Oligo(dT)_25_ (Invitrogen), 3 µl of Lysis/Binding Buffer (Invitrogen), and 0.25 pmol of each specific primer for the constant regions of rabbit γ and κ. The sequences of the primers were as follows: Igγ (5′-GCGAGTAGAGGCCTGAGGAC-3′) and Igκ (5′-GATGCCAGTTGTTTGGGTGGT-3′). The Dynabeads were then transferred into a solution containing 15 U of SuperScriptIII (Invitrogen), 1 U of murine RNase inhibitor (New England Biolabs), 0.5 mM of each dNTP, 5 mM DTT, 0.2% Triton X-100, and 1**×** First Strand Buffer (Invitrogen). A reverse transcription (RT) reaction was performed for 40 min at 50°C. After the RT reaction, the Dynabeads were transferred into another solution containing 20 U of terminal deoxynucleotidyl transferase (Roche), 0.5 mM dGTP, 1 U of murine RNase inhibitor, 4 mM MgCl_2_, 0.2% Triton-X 100, and 50 mM potassium buffer (25 mM K_2_HPO_4_ and 25 mM KH_2_PO_4_, pH 7.0), and incubated for 40 min at 37°C to add a poly-dG tail to the 3′ end of the cDNA. The Dynabeads were then transferred into a new PCR tube containing the first PCR reaction mix. The first PCR was performed using primeSTAR DNA polymerase (TaKaRa) according to the manufacturer’s instructions with a dC adaptor primer (5′-AGCAGTAGCAGCAGTTCGATAACTTCGAATTCTGCAGTCGACGGTACCGCGGGCCCGGGATCCCCCCCCCCCCCDN-3′) and a specific primer for the constant region of rabbit γ (Igγ-1st: 5′-CGAGTTCCAAGTCACGGTCA-3′) and rabbit κ (Igκ-1st: 5′-CTCCCAGGTGACGGTGACAT-3′). The PCR cycles were as follows: 5 min at 95°C followed by 30 cycles of 15 sec at 95°C, 5 sec at 55°C, and 1 min 30 sec at 72°C. The resultant PCR mixtures were diluted 4-fold with water, and 2 µl of the dilution was added to 23 µl of the nested PCR mix to serve as template DNA. The nested PCR was performed in a reaction mix similar to the first PCR mix using an adaptor primer (5′-AGCAGTAGCAGCAGTTCGATAA-3′) and a specific primer for the constant region of rabbit γ (Igγ-nest: 5′-GCCTTTGACCAAGCAGCCCAA-3′) or rabbit κ (Igκ-nest: 5′-CGGGAAAGTATTTATTCGCCACA-3′). The PCR cycles were as follows: 5 min at 95°C followed by 35 cycles of 15 sec at 95°C, 5 sec at 55°C, and 1 min 30 sec at 72°C. We inserted the PCR products into expression vectors that contained cDNAs for the whole constant region of rabbit γ or κ chains. Thereafter, we co-transfected CHO-S cells (Invitrogen) with both the γ and κchain expression vectors encoding whole antibody molecules using the FreeStyle MAX CHO Expression System (Invitrogen), and we collected the supernatants of cultured cells after 3 days. We examined the antigen specificity of the recombinant antibodies by ELISA and confirmed the results with competitive ELISA by adding soluble antigen to the antibodies [17], [36]. In this study, we screened only IgG-secreting cells with ISAAC. The immunoglobulin gene repertoire was analyzed with the IMGT/V-Quest tool (http://www.imgt.org/) [39]. For the determination of antibody affinity and western blotting, we collected the supernatants of cultured cells after 7 days and purified the RaMoAbs using a protein G column (GE Healthcare).

### Determination of Antibody Affinity

We determined antibody affinities (*K*D) at antibody-antigen equilibrium (Ab+Ag ↔ AbAg) in solution [40], [41]. Briefly, we incubated various concentrations (0.2, 1, or 10 nM) of HEL-specific monoclonal antibodies with 0.3 to 1,000 nM of HEL or 1 to 2,500 nM pTAK1-peptide overnight at 4°C until equilibrium was reached. We then measured the concentrations of free antibody that remained unsaturated with antigens at equilibrium by ELISA and calculated *K*D using Scatchard plot analysis. To this end, we coated 96-well MaxiSorp™ plates (Nunc) with 50 µl per well of 5 µg ml^−1^ HEL or 2.5 µg ml^−1^ pTAK1-peptide in PBS and subsequently blocked with 3% BSA in PBS. After washing, we added the mixture of antigen and antibodies at equilibrium to the plates and incubated for 1 h at room temperature. We detected antibodies that were unsaturated with antigens using IgG-specific antibodies conjugated to alkaline phosphatase and *p*-nitrophenylphosphate, and we measured the optical absorbance at 405 nm with an ELISA reader. From the optical absorbance, we calculated the concentration of antibody that was unsaturated with antigen [Abf] and the concentration of antigen that was not bound to antibody [Agf].

### Determination of LOD in ELISA

Ninety-six-well Maxisorp plates were coated with 50 µl per well of 1 µg ml^−1^ of IgG-specific antibody in PBS and then blocked with 3% BSA in PBS. After washing, 30 pM of the rabbit HEL-specific antibody was added to the plate and incubated for 1 hr at room temperature. The plate was washed with PBS-T three times, and various concentrations (100 to 0.1 pM) of biotinylated HEL were added to the plates and then incubated for 1 hr at room temperature. The binding of HEL was detected using 0.5 µg ml^−1^ of alkaline phosphatase labeled-streptavidin (Sigma) and *p*-nitrophenylphosphate. The optical absorbance was measured at 405 nm with an ELISA reader, according to the manufacturer’s instructions. LOD were determined by extrapolating the concentration from the signal equal to background signal plus 3 s.d.

### Western Blotting

We used a TAK1 antibody (M-579; Santa Cruz Biotechnology), a TAB1 antibody (C-20; Santa Cruz Biotechnology), and a phospho-TAK1 (Thr187) antibody (#4536; Cell Signaling Technology). We transfected HeLa cells with FLAG-tagged TAK1 and HA-tagged TAB1 expression plasmids [25] using Lipofectamine reagents (Invitrogen). We stimulated HeLa cells with 20 ng ml^−1^ TNF-α (R&D Systems) for 2 and 5 min. After the stimulation, we prepared whole cell lysates with lysis buffer (25 mM HEPES pH 7.7, 0.3 M NaCl, 1.5 mM MgCl_2_, 0.2 mM EDTA, 0.1% Triton X-100, 20 mM β-glycerophosphate, 1 mM sodium orthovanadate, 1 mM phenylmethylsulfonyl fluoride, 1 mM DTT, 10 µg ml^−1^ aprotinin, and 10 µg ml^−1^ leupeptin). We resolved the cell lysates by SDS-PAGE and transferred them to an Immobilon-P nylon membrane (Millipore). We treated the membrane with BlockAce (Dainippon Pharmaceutical Co.) and probed it with approximately 100 ng ml^−1^ of primary antibody as described above followed by horseradish peroxidase-conjugated anti rabbit or goat IgG-specific antibody (DAKO). Visualization was performed using the ECL system (GE Healthcare).

## Supporting Information

Figure S1 **Determination of affinity for HEL-specific RaMoAbs.** Various concentrations (0.2, 1, or 10 nM) of HEL-specific RaMoAbs were incubated with 0.3 to 1,000 nM HEL overnight at 4°C until equilibrium was reached. The concentration of free antibody that remained unsaturated at equilibrium was then measured by ELISA using HEL-coated 96-well plates. The concentration (nM) of HEL is indicated on the *x*-axis, and the absorbance at 405 nm is indicated on the *y*-axis. Data are representative of at least two independent experiments with similar results. The data were used for determination of *K*D using Scatchard plots.
(TIF)

Figure S2 **Determination of limit of detection of HEL in ELISA using rabbit and mouse HEL-specific antibodies.** Biotinylated HEL ranged from 100 pM to 0.1 pM were used to examine the limit of detection (LOD) of HEL using rabbit or mouse HEL-specific antibodies that showed the highest affinity among the obtained antibodies. LOD of the rabbit (Ra_HEL01; *K*D 2.63×10^−12^ M) (left) and mouse (*K*D 3.71×10^−10^ M) (right) HEL-specific antibodies were 0.4 pM and 10 pM, respectively. LOD was determined by extrapolating the concentration from the signal equal to background signal plus 3 s.d. of the background signal. Blue and red dotted lines indicate LODs of rabbit and mouse, respectively. Data are a representative of three independent experiments with similar results.
(TIF)

Figure S3 **Determination of affinity for pTAK1-peptide-specific RaMoAbs.** Various concentrations (0.2 or 6 nM) of pTAK1-peptide-specific RaMoAbs were incubated with 1 to 2,500 nM pTAK1-peptide overnight at 4°C until the equilibrium was reached. The concentration of free antibody that remains unsaturated at equilibrium was measured by ELISA using pTAK1-peptide-coated 96-well plate. The concentration (nM) of pTAK1-peptide is indicated on the *x*-axis, and the absorbance at 405 nm is indicated on the *y*-axis. Data are representative of at least two independent experiments with similar results. The data were used for determination of *K*D using Scatchard plots.
(TIF)

Table S1 **Efficiency of rabbit ISAAC for producing RaMoAbs.**
(XLS)
