# Supplementary figures and images for: A Novel Rabbit Immunospot Array Assay on a Chip Allows for the Rapid Generation of Rabbit Monoclonal Antibodies with High Affinity
Source: PLoS One. 2012 Dec 26;7(12):e52383. doi: 10.1371/journal.pone.0052383 (PMC3530603; doi:10.1371/journal.pone.0052383)

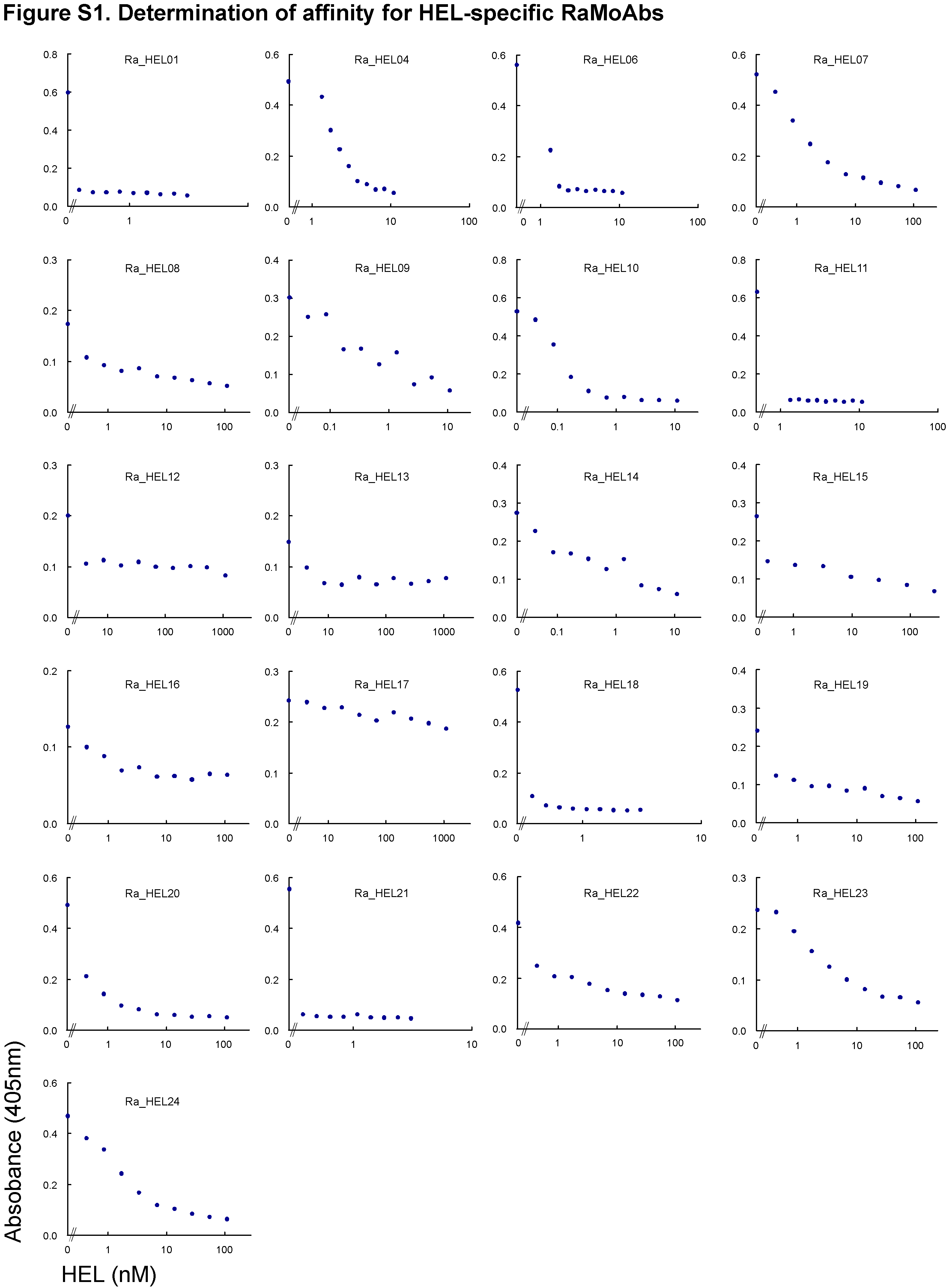

Supplement: Figure S1 — Determination of affinity for HEL-specific RaMoAbs. Various concentrations (0.2, 1, or 10 nM) of HEL-specific RaMoAbs were incubated with 0.3 to 1,000 nM HEL overnight at 4°C until equilibrium was reached. The concentration of free antibody that remained unsaturated at equilibrium was then measured by ELISA using HEL-coated 96-well plates. The concentration (nM) of HEL is indicated on the x-axis, and the absorbance at 405 nm is indicated on the y-axis. Data are representative of at least two independent experiments with similar results. The data were used for determination of KD using Scatchard plots. (TIF) [file pone.0052383.s001.tif]

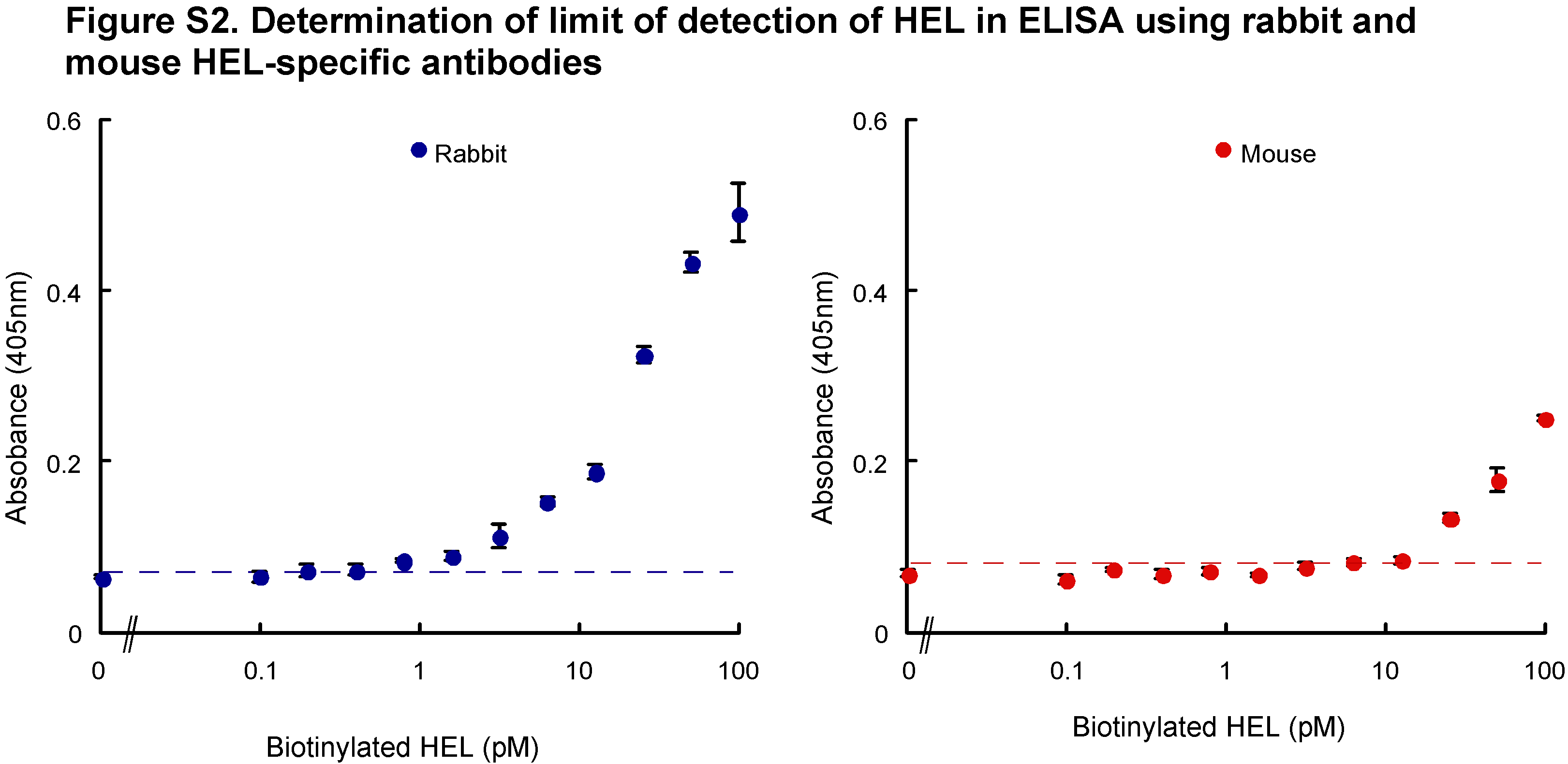

Supplement: Figure S2 — Determination of limit of detection of HEL in ELISA using rabbit and mouse HEL-specific antibodies. Biotinylated HEL ranged from 100 pM to 0.1 pM were used to examine the limit of detection (LOD) of HEL using rabbit or mouse HEL-specific antibodies that showed the highest affinity among the obtained antibodies. LOD of the rabbit (Ra_HEL01; KD 2.63×10^−12^ M) (left) and mouse (KD 3.71×10^−10^ M) (right) HEL-specific antibodies were 0.4 pM and 10 pM, respectively. LOD was determined by extrapolating the concentration from the signal equal to background signal plus 3 s.d. of the background signal. Blue and red dotted lines indicate LODs of rabbit and mouse, respectively. Data are a representative of three independent experiments with similar results. (TIF) [file pone.0052383.s002.tif]

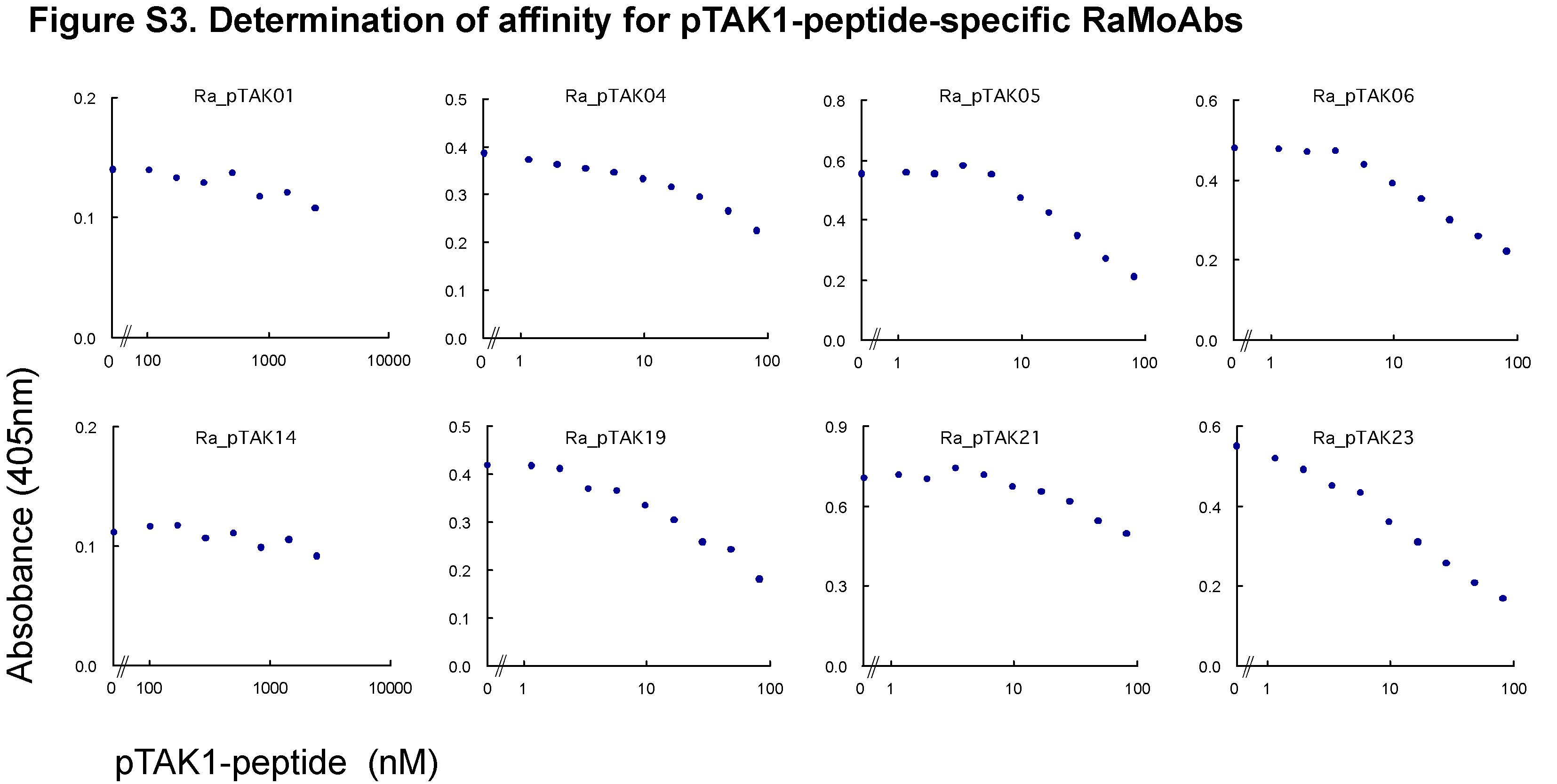

Supplement: Figure S3 — Determination of affinity for pTAK1-peptide-specific RaMoAbs. Various concentrations (0.2 or 6 nM) of pTAK1-peptide-specific RaMoAbs were incubated with 1 to 2,500 nM pTAK1-peptide overnight at 4°C until the equilibrium was reached. The concentration of free antibody that remains unsaturated at equilibrium was measured by ELISA using pTAK1-peptide-coated 96-well plate. The concentration (nM) of pTAK1-peptide is indicated on the x-axis, and the absorbance at 405 nm is indicated on the y-axis. Data are representative of at least two independent experiments with similar results. The data were used for determination of KD using Scatchard plots. (TIF) [file pone.0052383.s003.tif]
